# Elevations in Tumor Necrosis Factor Alpha and Interleukin 6 From Neuronal-Derived Extracellular Vesicles in Repeated Low-Level Blast Exposed Personnel

**DOI:** 10.3389/fneur.2022.723923

**Published:** 2022-04-21

**Authors:** Katie A. Edwards, Jacqueline J. Leete, Ethan G. Smith, Alycia Quick, Claire M. Modica, Eric M. Wassermann, Elena Polejaeva, Kristine C. Dell, Matthew LoPresti, Peter Walker, Meghan O'Brien, Chen Lai, Bao-Xi Qu, Christina Devoto, Walter Carr, James R. Stone, Stephen T. Ahlers, Jessica M. Gill

**Affiliations:** ^1^Biomarkers of Trauma, National Institute of Nursing Research, National Institutes of Health, Bethesda, MD, United States; ^2^Henry M. Jackson Foundation for the Advancement of Military Medicine, Bethesda, MD, United States; ^3^School of Psychology, University of Glasgow, Glasgow, United Kingdom; ^4^Naval Medical Research Center, Silver Spring, MD, United States; ^5^National Institute of Neurological Disorders and Stroke, National Institutes of Health, Bethesda, MD, United States; ^6^VA San Diego Healthcare System, San Diego, CA, United States; ^7^Department of Psychology, Pennsylvania State University, University Park, PA, United States; ^8^Center for Military Psychiatry and Neuroscience, Walter Reed Army Institute of Research, Silver Spring, MD, United States; ^9^Joint Artificial Intelligence Center, Arlington, VA, United States; ^10^Department of Radiology and Medical Imaging, University of Virginia, Charlottesville, VA, United States; ^11^Oak Ridge Institute for Science and Education, Oak Ridge, TN, United States; ^12^Naval Medical Research Center, Operational and Undersea Medicine Directorate, Silver Spring, MD, United States; ^13^Center for Neuroscience and Regenerative Medicine, Uniformed Services of the Health Sciences, Bethesda, MD, United States

**Keywords:** extracellular vesicles, neuroinflammation, breacher, blast, military

## Abstract

**Objective:**

The purpose of this pilot study was to determine if military service members with histories of hundreds to thousands of low-level blast exposures (i. e., experienced breachers) had different levels of serum and neuronal-derived extracellular vesicle (EV) concentrations of interleukin (IL)-6, IL-10, and tumor necrosis factor alpha (TNFα), compared to matched controls, and if these biomarkers related to neurobehavioral symptoms.

**Methods:**

Participants were experienced breachers (*n* = 20) and matched controls without blast exposures (*n* = 14). Neuronal-derived EVs were isolated from serum and identified with mouse anti-human CD171. Serum and neuronal-derived EVs were analyzed for IL-6, IL-10, and TNFα using an ultra-sensitive assay.

**Results:**

Serum TNFα concentrations were decreased in breachers when compared to control concentrations (*p* < 0.01). There were no differences in serum concentrations of IL-6, IL-10, or the IL-6/IL-10 ratio between breachers and controls (*p*'s > 0.01). In neuronal-derived EVs, TNFα and IL-6 levels were increased in breachers compared to controls (*p*'s < 0.01), and IL-10 levels were decreased in the breacher group compared to controls (*p* < 0.01). In breachers the IL-6/IL-10 ratio in neuronal-derived EVs was higher compared to controls, which correlated with higher total Rivermead Post-concussion Questionnaire (RPQ) scores (*p*'s < 0.05).

**Conclusions:**

These findings suggest that exposure of personnel to high numbers of low-level blast over a career may result in enduring central inflammation that is associated with chronic neurological symptoms. The data also suggest that peripheral markers of inflammation are not necessarily adequate surrogates for central neuroinflammation.

## Introduction

Military personnel and law enforcement breachers represent a unique population who experience repetitive, low-level blast exposures during training and occupational duties, often over a prolonged period (1). While these low-level exposures do not result in a diagnosed concussion or traumatic brain injury (TBI), the cumulative effect of these exposures may be linked to cognitive and behavioral symptoms (2). Previously, we have reported elevated serum cytokines following acute exposure to blast from military personnel in close temporal proximity to multiple blast events of varying intensities (3). In this study, we turn our attention to the assessment of potential long-term manifestations in a population of personnel exposed to hundreds to thousands of blast events over a career and with no acute exposure to understand the cumulative effect of repetitive blast on central inflammation which may be associated with cognitive and neurobehavioral symptoms in a manner similar to military personnel with diagnosed TBIs (4, 5).

Extracellular vesicles (EVs) are formed within cells and can transfer cargo by binding to other cells and releasing the cargo, such as cytokines, altering cellular activity, including inflammatory pathways. Neuronally-derived EVs can be isolated from blood, and thus provide insights into central cell-to-cell activity (6, 7). Several studies have found exosomal changes in biomarkers isolated from serum after TBI (8–10). Recently, higher levels of interleukin (IL)-6 were reported in neuronal-derived exosomes of Veterans with multiple mild TBIs and chronic neurological symptoms, including possible blast exposures (10), suggesting central inflammatory activities relate to mild TBIs. However, the impact of exposure to a high number of low-level blast exposures over a prolonged career on central inflammation remains to be determined. The purpose of this pilot study was to examine relationships of serum and neuronal-derived EV concentrations of IL-6, IL-10, and tumor necrosis factor alpha (TNFα) with repetitive, low-level blast exposures and neurological symptoms in an experienced breacher population and a cohort of well-matched control subjects. We hypothesize that EV inflammatory markers correlate with neurologic effects from repetitive, low level blast exposure and that expression of EV inflammatory markers differ from serum inflammatory markers.

## Materials and Methods

### Standard Protocol Approvals, Registrations, and Subject Consents

The National Institutes of Health (NIH) and Naval Medical Research Center (NMRC) Institutional Review Boards granted approval for this study. Participants provided written informed consent prior to enrolling in the study for all procedures performed.

### Methodology

Participants in this study included 20 current or former active duty military or civilian law enforcement breachers. Breaching involves the use of explosives to gain entry to hardened structures and military and law enforcement populations involved with breaching over a career are exposed to potentially hundreds of explosive events in the course of training and operations. Inclusion criteria for breachers consisted of at least 4 years of experience in the breaching profession and active involvement (at least annually) in training or operations related to breaching. Former breachers qualified for the study with a self-reported history of exposure to 400 or more breaching blasts within their career. A total of 14 matched controls were enrolled in the study who were matched in age, gender, and operational experience to the breacher group. Controls must have had at least 4 years of experience in the military or civilian law enforcement profession, be actively involved in military or civilian law enforcement training or operations and have a blast exposure history of fewer than 40 individual blasts over their career. Exclusion criteria for both breachers and controls included a history of moderate to severe brain injury with loss of consciousness over 5 min or a central nervous system, cardiac, respiratory condition or other medical condition known to impact cerebral metabolism.

### Demographics, Clinical History, and Psychometric Testing

Assessments were performed on the NIH Campus, which was part of a larger effort to understand clinical impacts of blast exposures. A description of the full battery of neurological and functional tests, neuroimaging protocols, and biomarker analysis is described elsewhere (11), as well as overall study findings. During the evaluation, interviews were conducted to collect demographics and clinical history. A battery of psychometric tests was administered, including the Posttraumatic Stress Disorder Checklist—Military version (PCL-M) and the Rivermead Postconcussion Questionnaire (RPQ) used for analysis in this study. The PCL-M is a self-report measure that assesses 20 symptoms of post-traumatic stress disorder (PTSD) as identified in the DSM-IV (12). The RPQ consists of 16 items measuring the extent of physical, cognitive, and behavioral post-concussive symptoms compared to before the injury (13). Each item is rated on a 5-point ordinal scale from 0 (symptom not experienced) to 4 (severe), with a total score of 0–64 (least to most symptomatic). The modified RPQ further breaks down the questionnaire into physical and cognitive/behavioral symptom clusters (14). The RPQ-3 measures 3 items in the physical symptoms cluster (headaches, feelings of dizziness, nausea and/or vomiting) with a total score of 0–12 (best to worst). The RPQ-3 physical symptoms cluster tends to be associated with early post-concussive symptoms immediately following injury. The RPQ-13 measures 13 items in the cognitive/behavioral symptom cluster (noise sensitivity, sleep disturbance, fatigue, irritability, depression, frustration, forgetfulness, poor concentration, taking longer to think, blurred vision, light sensitivity, double vision, and restlessness), with a total score of 0–52 (best to worst). The RPQ-13 cognitive/behavioral symptoms cluster is associated with late post-concussive symptoms occurring in the days and weeks following injury, although RPQ-3 physical symptoms may continue into this late stage as well.

### Blood Biomarkers

Non-fasting blood samples were processed for serum within 1 h using standard protocols (15) between 9:00 am and 12:00 pm prior to interviews and the psychometric testing, and stored at −80°C until batch assay processing.

### Neuronal-Derived EV Isolation

EVs were isolated from thawed samples as previously published (10). Briefly, thawed samples were centrifuged at 3,000 × g for 15 min to remove cells and cell debris. The supernatant was added with an appropriate volume of ExoQuick reagent, mixed well and incubated 60 min at + 4°C. The ExoQuick/sample mixture was then centrifuged at 1,500 × g for 30 min. The resulting pellet containing all EVs was resuspended in equal volume of 1 x PBS.

To isolate neuronal-specific EVs, 50–100 μL of 3% BSA in 1 x PBS was added to the EV suspension with biotinylated mouse anti-human CD171 [L1 cell adhesion molecule (L1CAM)] biotinylated antibody (clone 5G3, eBioscience, San Diego, CA) and incubated for 60 min at 4°C, followed by addition of 15 μl of streptavidin-agarose Ultralink resin (Thermo Scientific, Inc.) in 40 μL of 3% BSA and incubation for 30 min at 4°C with continuous mixing. After centrifugation at 200 × g for 10 min at 4°C and removal of the supernatant, the resin pellet was washed 3 times with PBS. Each pellet was re-suspended in equal volume of Ab/Ag dissociation buffer (Pierce™ Gentle Ag/Ab Elution Buffer 21,027, pH 6.6) by mixing for 10 s and centrifuged at 4,500 × g for 10 min at 40°C. Supernatants were transferred to clean tubes for further testing. EVs from breacher and control serum samples were characterized using MACSplex assay, and FlowLogic and MACSQuant softwares (Miltenyi Biotec) were used to analyze flow cytometric data, as described in detail previously (16).

### Cytokine Quantification

Serum samples and isolated neuronal-derived EVs were removed from the −80°C freezer and thawed on ice. EVs were lysed with equal volume of mammalian protein extraction reagent (M-PER) (Thermo-Fisher Scientific Inc., Rockford, IL, USA) with cOmplete™ Protease Inhibitor Cocktail (Roche). All proteomic analyses were measured using the high-definition (HD-1), single-molecule array technology (SIMOA™, Quanterix, Lexington, MA). Serum samples were analyzed for cytokines: IL-6, IL-10 and TNFα (Simoa Cytokine 3-Plex Advantage Kit, Quanterix, Lexington, MA) using the method previously described by Rissin et al. (17). The Simoa™ analyzer runs ultra-sensitive, paramagnetic digital, enzyme-linked immunosorbent assays and analyzes each sample in duplicate (18). The coefficient of variation for all concentration values were <20%. The lower limit of detection (LLoD) for each assay are as follows: IL-6, 0.006 pg/mL; IL-10, 0.0022 pg/mL; TNFα, 0.011 pg/mL. Values below LLoD were excluded from analyses.

### Statistical Analysis

Statistical testing was conducted with IBM SPSS Build 1.0.0.1298 (Armonk, NY: IBM Corp.). GraphPad Prism version 8 (La Jolla, CA: GraphPad Software) was used to create figures. Clinical and demographic differences were calculated using chi square and independent samples *t*-test. Biomarker concentrations were first natural log-transformed (ln) prior to comparing the two groups (breachers and controls) with one-way ANOVA; presented results include mean (SD) and F-statistic with *p*-value for significant results. Graphical representations of log-transformed biomarker concentrations include horizontal lines for mean and standard deviation values. Within the breacher group, Pearson correlations were conducted to determine the relationship between RPQ scores and log-transformed biomarker concentrations; significant values are presented as graphs including line of best fit.

## Results

### Demographic and Clinical Characteristics

There were 34 male participants included in the study (20 breachers and 14 controls) with a mean age of 39 years. The majority of participants were white (85%) military personnel (65–71%). No differences in age, education, or prior service were observed between the breacher and control groups (Table 1). While we found a significant difference in the number of head injuries, this number was self-reported. Participants were excluded for moderate or severe TBI. Breachers had higher scores on the PCL-M and on the RPQ-3 section of the Rivermead Postconcussive Questionnaire (Table 1).

**Table 1:** Demographic and clinical characteristics.

	**Breacher (*N* = 20)**	**Matched controls (*N* = 14)**	**Significance**
**Race, no. (%)**			χ^2^ = 2.893, *p* = 0.576
White	17 (85)	12 (85.71)	
Black	0 (0)	1 (7.14)	
Asian/pacific islander	1 (5)	1 (7.14)	
American Indian/Alaskan	1 (5)	0 (0)	
Other	1 (5)	0 (0)	
Ethnicity (Non-Hispanic), no. (%)	19 (95)	13 (92.86)	χ^2^ = 0.068, *p* = 0.794
Sex (male), no. (%)	20 (100)	14 (100)	N/A
Type of service, no. (%)			χ^2^ = 0.336, *p* = 0.562
Military	13 (65)	10 (71.43)	
Civilian law enforcement	4 (20)	4 (28.57)	
Both	3 (15)	0 (0)	
Mean age in years (SD)	39.65 (8.337)	38.86 (7.814)	*t* = 0.280, *p* = 0.781
Mean years of education (SD)	14.25 (1.743)	14.43 (2.593)	*t* = −0.241, *p* = 0.811
Mean years of service (SD)	16.80 (6.693)	13.92 (6.986)	*t* = 1.209, *p* = 0.235
Most recent blast exposure, no. (%)			χ^2^ = 29.046, *p* < 0.01
Never	0 (0)	11 (78.57)	
Past week	4 (20)	0 (0)	
Past month	8 (40)	0 (0)	
Past 6 months	3 (15)	0 (0)	
Past year	3 (15)	0 (0)	
More than 1 year	2 (5)	3 (21.43)	
Mean number of self-reported head injuries (SD)	0.80 (0.616)	0.36 (0.497)	*t* = 2.228, *p* = 0.033
PCL-M	25.55 (6.924)	20.64 (4.483)	*t* = 2.327, *p* = 0.027
**RPQ scores**			
Total RPQ	11.95 (11.928)	6.93 (6.158)	*t* = 1.436, *p* = 0.161
RPQ-3	1.53 (2.065)	0.21 (0.426)	*t* = 2.332, *p* = 0.026
RPQ-13	10.42 (10.772)	6.71 (6.207)	*t* = 1.151, *p* = 0.258

### Serum and EV Cytokine Results

**Figure 1:**
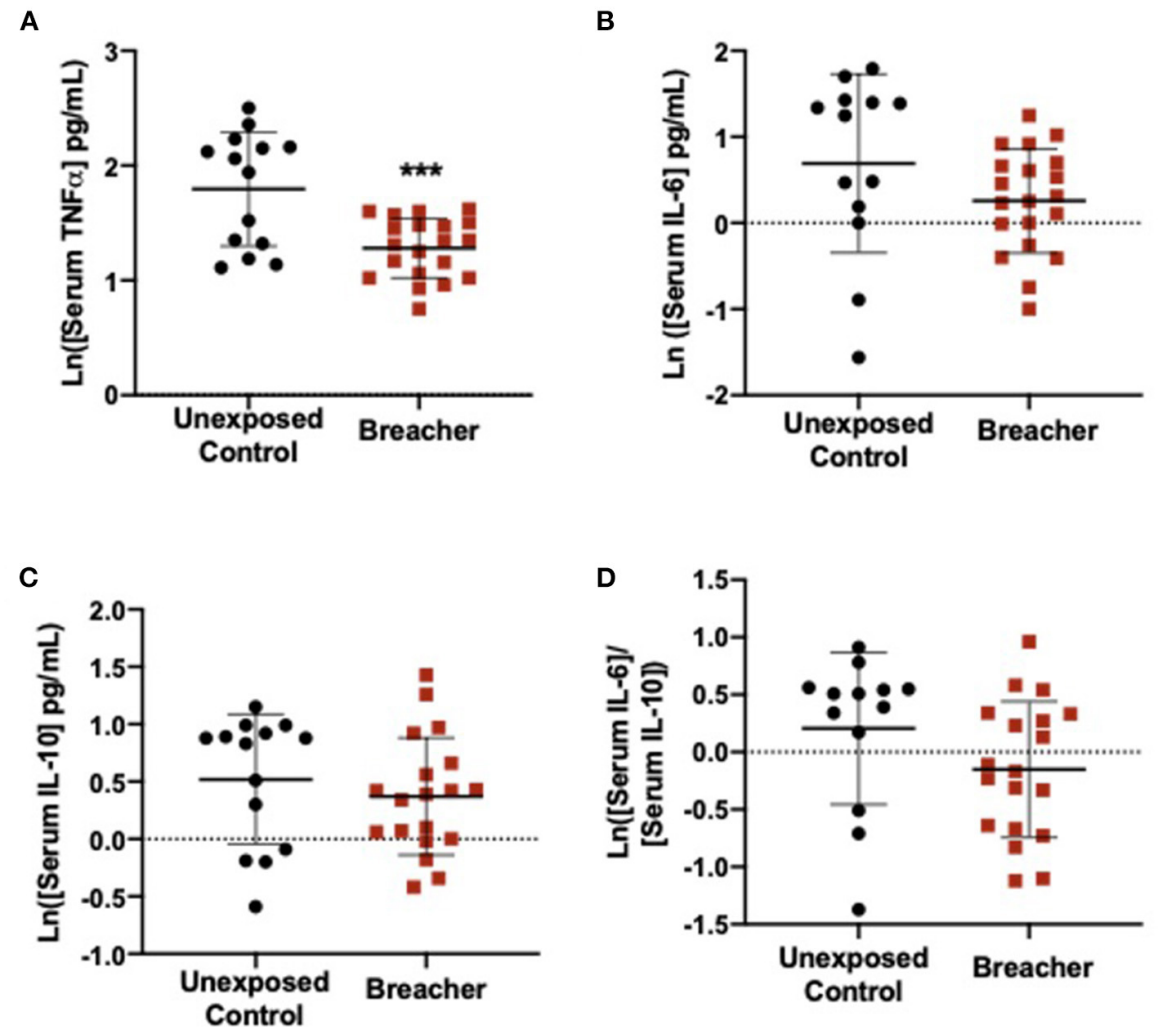
Serum levels of pro-inflammatory cytokine TNFα decreased in breachers. **(A)** Serum TNFα is decreased in breachers as compared to unexposed controls, **(B)** No change in serum IL-6 in breachers as compared to unexposed controls, **(C)** No change in serum IL-10 between groups, **(D)** No change in the IL-6/IL-10 ratio between groups. Significant *p*-values are represented as: ***p < 0.001.

**Figure 2:**
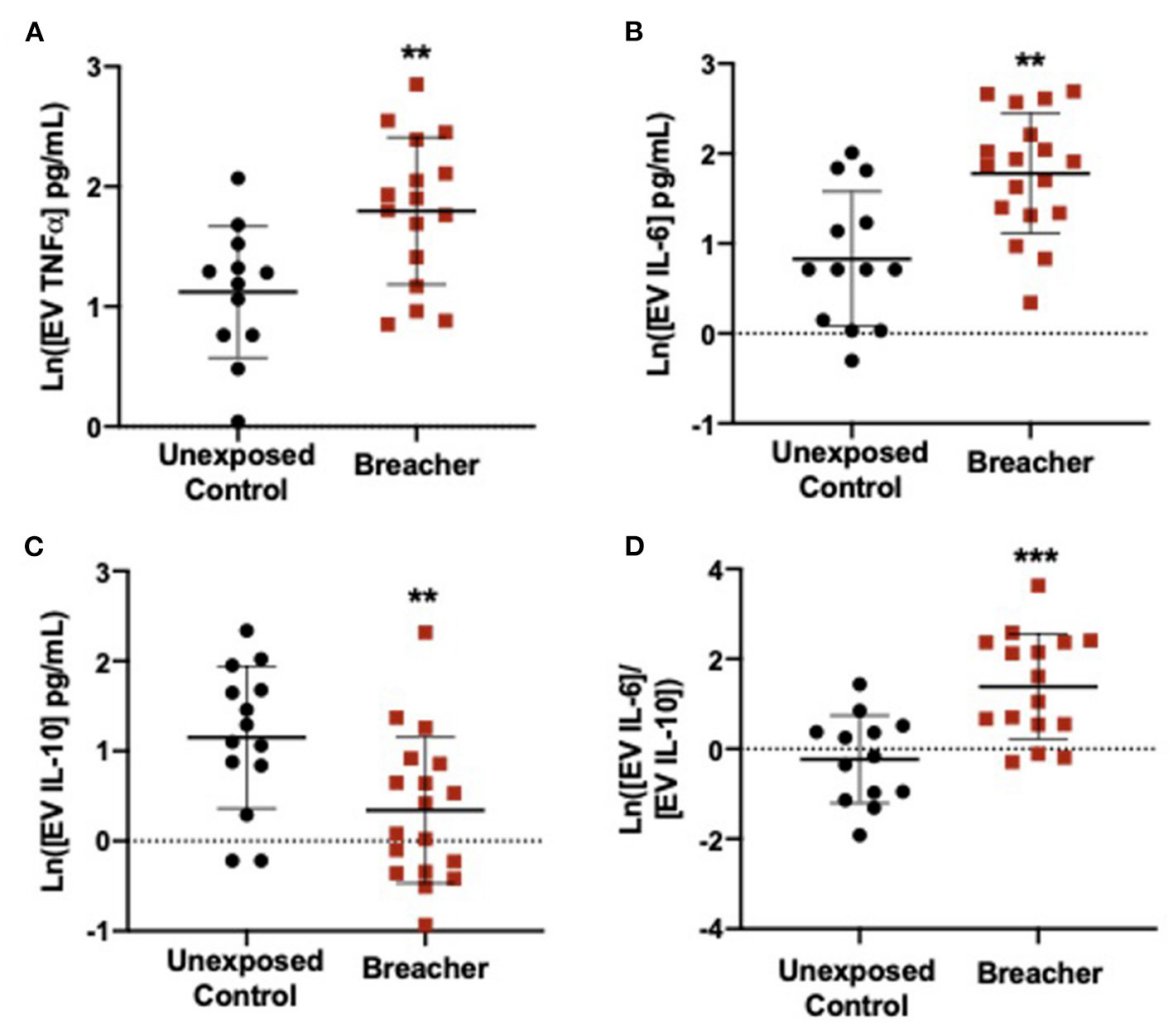
Neuronal-derived EV cytokines propagate a pro-inflammatory environment in the brain. **(A,B)** EVs derived from neurons contain elevated levels of pro-inflammatory cytokines TNFα and IL-6 in experienced breachers when compared to unexposed controls. In addition, **(C)** anti-inflammatory cytokine IL-10 is decreased in this population. **(D)** The ratio of pro/anti-inflammatory cytokines IL-6/IL-10 is higher in experienced breachers. Significant *p*-values are represented as: **p < 0.01, ***p < 0.001.

In the breacher group, the log-transformed (ln) TNFα concentrations in serum were decreased in breachers (1.281 ± 0.2601) when compared to controls (1.797 ± 0.4972) (*F* = 15.574, *p* = 0.0004) (Figure 1A), with no differences in serum concentrations of IL-6 (*p* = 0.137), IL-10 (*p* = 0.437), or the IL-6/IL-10 ratio (*p* = 0.121) between these groups (Figures 1B–D). Concentrations of neuronal EV-specific TNFα was increased in breachers (1.797 ± 0.6143) compared to controls (1.120 ± 0.5504) (*F* = 9.092, *p* = 0.006) (Figure 2A), as well as IL-6 (1.780 ± 0.6651) vs. (0.829 ± 0.7484) (*F* = 13.919, *p* = 0.001) (Figure 2B). However, EV-specific IL-10 concentrations were decreased in the breachers (0.343 ± 0.8123) compared to controls (1.150 ± 0.7900) (*F* = 7.970*, p* = 0.008) (Figure 2C). Lastly, there was a higher IL-6/IL-10 ratio in neuronal-derived EVs in the breachers (1.3484 ± 1.1737) when compared to the controls (−0.230 ± 0.9758) (*F* = 15.772, *p* = 0.0005) (Figure 2D).

### Cytokines Relation to Post-concussive Disorder and PTSD Symptoms With Breachers

**Figure 3:**
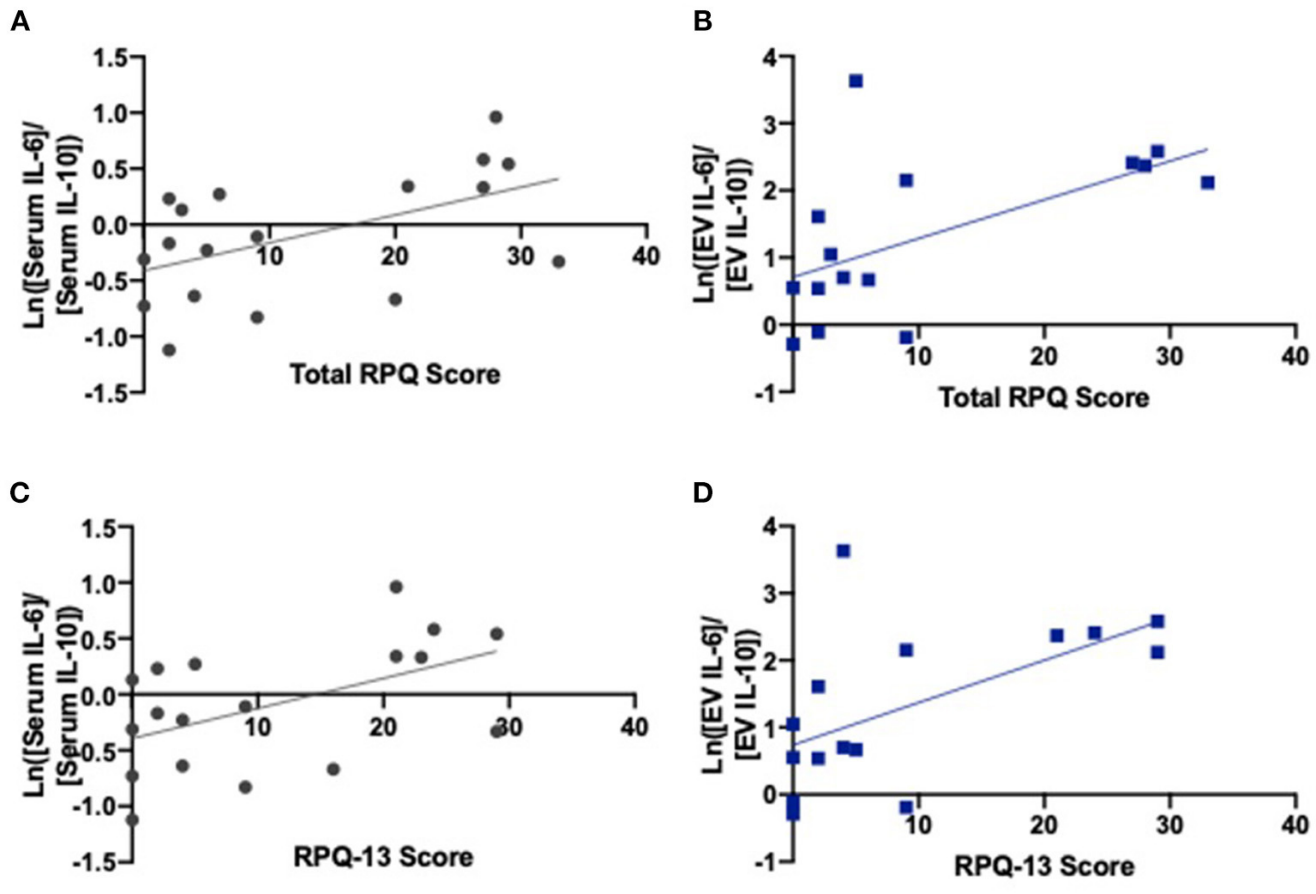
IL-6/IL-10 ratios correlate with late postconcussive symptoms. **(A)** Serum ratios of IL-6/IL-10 (*r* = 0.526, *p* = 0.025) and **(B)** EV ratios of IL-6/IL-10 correlate with total RPQ score (*r* = 0.585, *p* = 0.022). **(C)** Serum ratios of IL-6/IL-10 (*r* = 0.515, *p* = 0.029) and **(D)** EV ratios of IL-6/IL-10 correlate with RPQ-13 scores (*r* = 0.580, *p* = 0.023), the portion of the exam related to late postconcussive symptoms. Graphs include line of best fit.

We found correlations between higher total RPQ scores and increased serum IL-6/IL-10 ratios (*r* = 0.526, *p* = 0.025) as well as increased neuronal-derived EV IL-6/IL-10 ratios (*r* = 0.585, *p* = 0.022) (Figure 3; Table 2). We also identified correlations between higher RPQ-13 scores and increased IL-6/IL-10 ratios from serum (*r* = 0.515, *p* = 0.029) and EVs (*r* = 0.580, *p* = 0.023). There were no significant relationships between PTSD symptoms and cytokine concentrations.

**Table 2:** Pearson correlations of log-transformed biomarkers with PCL-M and RPQ scores.

		**Scales**			
		**PCL-M**	**Total RPQ**	**RPQ-3**	**RPQ-13**
Serum TNFα	Pearson correlation	−0.103	0.209	0.137	0.205
	Sig. (2-tailed)	0.666	0.390	0.575	0.400
	*N*	20	19	19	19
Serum IL-6	Pearson correlation	−0.015	0.085	0.184	0.059
	Sig. (2-tailed)	0.949	0.728	0.451	0.809
	*N*	20	19	19	19
Serum IL-10	Pearson correlation	−0.144	−0.403	−0.115	−0.423
	Sig. (2-tailed)	0.556	0.097	0.649	0.080
	*N*	19	18	18	18
Serum IL-6/IL-10	Pearson correlation	0.140	0.526^*^	0.339	0.515^*^
	Sig. (2-tailed)	0.566	0.025	0.168	0.029
	*N*	19	18	18	18
Neuronal-derived EV TNFα	Pearson correlation	0.097	−0.213	−0.126	−0.216
	Sig. (2-tailed)	0.720	0.446	0.655	0.439
	N	16	15	15	15
Neuronal-derived EV IL-6	Pearson correlation	0.135	0.227	−0.010	0.249
	Sig. (2-tailed)	0.593	0.382	0.968	0.335
	*N*	18	17	17	17
Neuronal-derived EV IL-10	Pearson correlation	−0.149	−0.478	−0.378	−0.461
	Sig. (2-tailed)	0.556	0.052	0.134	0.063
	*N*	18	17	17	17
Neuronal-derived EV IL-6/IL-10	Pearson correlation	0.205	0.585^*^	0.346	0.580^*^
	Sig. (2-tailed)	0.445	0.022	0.207	0.023
	N	16	15	15	15

* *indicates significant correlation (2-tailed)*.

## Discussion

In this pilot study we report significantly higher concentrations inflammatory cytokines TNFα and IL-6 in neuronal-derived EVs, whereas concentrations of IL-10 were lower in experienced breachers compared to controls. Ratios of IL-6 and IL-10 were significantly different in breachers' neuronal-derived EVs compared to controls and that these ratios relate to post-concussive disorder symptoms, suggesting dysregulation of immune activity may relate to high blast exposures and place individuals at greater risk for chronic symptoms. Unexpectantly, we also report that serum levels of TNFα were reduced in breachers compared to controls within the context of higher concentrations in neuronal derived EVs, suggesting that inflammation may be related to central processes. These findings suggest a role for inflammatory cytokines in the effects of cumulative repetitive, low-level blast exposures and suggest that these biomarker changes relate to chronic symptoms.

Our current finding of lower serum levels of TNFα in the breacher group differs from our previous work in which we observed an increase in serum TNFα and IL-6 in breachers undergoing blast exposure in an acute setting (3). This discrepancy suggests that a career of high blast exposures may result in differential immune activity from that of acute exposure. Our findings do align with a recent report in a population of military personnel with blast TBI and non-acute serum sampling, in which pro-inflammatory markers IL-8 and MMP3 measured in serum were significantly decreased and IL-6 followed a similar decreasing pattern, though it did not reach significance (19). While previous studies have measured IL6, IL10, and TNFα from serum in breachers (3), our study is the first description of these inflammatory cytokines in neuronal-derived EVs from a unique cohort of experienced breachers.

Our data suggest that peripheral levels of circulating cytokines may not be as informative of neuroinflammatory activity which is observed in neuronal derived EV concentration of cytokines. Injury to the brain often compromises the blood-brain barrier and also activates a secondary cascade where microglia and astrocytes release a variety of factors that can damage neurons and prolong the inflammatory response within the brain for months or years (20, 21). Importantly, secondary damage to the brain may lead to chronic neuroinflammatory imbalance and continue to have adverse effects on neurological function after the primary damage has resolved (22). Thus, the ability to distinguish CNS from peripheral inflammatory response is crucial for a better understanding of how the inflammatory response may impact neurological outcomes long-term.

Notably, proteins within EVs are protected from proteinase-specific degradation, meaning EV-isolated cytokine levels are potentially more stable than those in serum and may be further suitable for clinical applications (23). Literature specific to EVs in blast exposure is limited, however, studies in other neurological processes such as TBI have successfully assessed the total content of EVs isolated from blood in military populations (24–27). For example, differential expression of EV miRNA related to inflammatory and neuronal repair pathways is observed in veterans with remote blast-related mTBI (27), supporting protein findings. EVs isolated from saliva show differential inflammatory gene expression in civilian populations with acute head trauma (28, 29). While these studies examine total EVs from blood and saliva, neuronal cell specific proteins can also be identified in total EVs isolated from cerebral spinal fluid (CSF) (30). In most contexts where use of CSF is not feasible, the isolation of neuronal-derived EVs provides enhanced specificity to the CNS processes. A sports-related mild TBI study found that protein concentrations within neuronal-derived EVs differed depending on the time since injury, including elevations in IL-6 (31). Neuronal-derived EV proteins have been shown to differ depending on cognitive impairment status, including IL-6, in veterans with remote TBI (32, 33). In addition, work is expanding to include the role of EVs from other brain parenchymal cells beyond neurons, including astrocytes, microglia, and oligodendrocytes (8, 34, 35). Future studies specific to breacher populations are warranted, such as measuring miRNA and distinguishing cytokine profiles in EVs from astrocytes and microglia.

The cytokine levels isolated from neuronal-derived EVs differed from our observations of serum cytokine levels. In breachers, we found elevated levels of IL-6 and TNFα in neuronal-derived EVs. In addition, we identified lower levels of IL-10 from neuronal-derived EVs. The balance between the pro- and anti-inflammatory activity is essential, as it creates an environment essential for neuronal health, and a proinflammatory environment has been associated with risk for the development of neurodegenerative diseases (36) and has been shown to influence cognitive behavior (37, 38). Therefore, we quantified the ratio between IL-6 and IL-10; if pro-inflammatory cytokines are upregulated compared to anti-inflammatory cytokines, or if anti-inflammatory cytokines are down-regulated, the ratio of IL-6/IL-10 will be larger. IL-6/IL-10 ratios have been used as a predictive marker for disease severity (39), but few have investigated their potential in predicting long-term health after blast exposures. Breachers had higher EV-specific IL-6/IL-10 ratios, implying the pro-inflammatory environment after repetitive, low-level blast exposures may be attributed to the chronic neuroinflammatory imbalance found in NF-κB pathway activation and reduced neurologic outcome.

Particularly, higher IL-6/IL-10 ratios in breachers correlated with higher total RPQ scores as well as RPQ-13 scores in both serum and EVs, implying that breachers who have inflammatory activity in the periphery, and possibly in the CNS, increase the risk for more chronic symptoms. However, we found no increased inflammatory response in the periphery of breachers compared to controls, highlighting the complications in utilizing peripheral cytokine levels alone to determine personnel most at risk for developing long-term symptoms. Regardless, our findings continue to suggest that neuronal-derived cytokines may contribute to a pro-inflammatory environment in the brain that could impact cognitive function by influencing injury-related processes in the CNS. Therefore, additional studies to further determine these relationships are warranted.

This study includes several strengths, including the isolation of neuronal-derived EVs from serum and the observation of increased CNS inflammation associated with neurological symptoms in a unique breacher population. This study was limited by the small sample size and the use of one time-point, preventing us from verifying the clinical significance of neuroinflammatory proteins in predicting adverse symptoms in breachers. The number of breaching exposures was estimated by participants and subject to recall bias. In addition, symptoms presented in this study are primarily self-reported and do not include any confirmation of reported symptoms through extensive testing. Although previous work suggests that certain EV cytokines (including IL-6, IL-10, and TNFα), are primarily encapsulated within EVs (40), distinguishing between EV surface-level and encapsulated cytokines was outside the scope of this study. Identification of the mechanism leading to the observed increase in inflammatory cytokines in circulating neuronal-derived EVs was also outside the scope of this study. However, these results show the importance of examining links between inflammation and neurological symptoms and call for further studies specific to experienced career breachers.

Overall, the findings suggest CNS-specific inflammatory responses following low-level blast exposures may be related to chronic symptoms. Future studies identifying biomarkers in circulating blood as well as proteins isolated from neuronal-derived EVs are warranted. In addition, research should examine whether neuroinflammatory biomarkers influence the development of unfavorable outcomes in breachers.

## Data Availability Statement

The raw data supporting the conclusions of this article will be made available by the authors, without undue reservation.

## Ethics Statement

The studies involving human participants were reviewed and approved by the National Institutes of Health (NIH) and Naval Medical Research Center (NMRC) Institutional Review Boards. The patients/participants provided their written informed consent to participate in this study.

## Author Contributions

KE designed and conceptualized the study, analyzed the data, and revised the manuscript for intellectual content. JL, ES, and EW contributed substantial revisions to the manuscript. AQ and CM had a major role in the acquisition of the data and contributed the analysis of the data. EP, KD, ML, PW, and WC managed and executed the protocol, recruited the subjects, collected the samples, and contributed to the collection of data. MO'B managed and executed the protocol and contributed to the management of data. CL, B-XQ, and CD had a major role in the acquisition of the data. JS contributed substantial revisions to the manuscript, study supervision, and coordination. SA contributed study supervision and coordination, obtained funding, and contributed substantial revisions to the manuscript. JG contributed to the conception and design of the study and revised the manuscript for intellectual content. All authors contributed to the article and approved the submitted version.

## Funding

This work was funded by the Joint Program Committee-5 Development of Exposure Standards to Repeated Blast Exposure program, work unit #603115HP-3730-001-A1118. This work was also supported by National Institute of Nursing Research (NINR) and National Institute of Neurological Disease and Stroke (NINDS) Intramural Research Programs. This work was supported in part by an appointment to the Research Participation Program at the Walter Reed Army Institute of Research administered by the Oak Ridge Institute for Science and Education through an interagency agreement between the U.S. Department of Energy and the U.S. Army Medical Research and Development Command. Some of the authors are Military Service Members (or employees of the U.S. Government). This work was prepared as part of their official duties. Title 17, U.S.C., §105 provides that copyright protection under this title is not available for any work of the U.S. Government. Title 17, U.S.C., §101 defines a U.S. Government work as a work prepared by a Military Service Member or employee of the U.S. Government as part of that person's official duties.

## Author Disclaimer

The views expressed in this manuscript reflect the results of research conducted by the authors and do not necessarily reflect the official policy or position of the Department of the Army, the Department of the Navy, Department of Defense, or the United States Government. Material has been reviewed by the Walter Reed Army Institute of Research. There is no objection to its presentation and/or publication. The investigators have adhered to the policies for protection of human subjects as prescribed in AR 70-25.

## Conflict of Interest

The authors declare that the research was conducted in the absence of any commercial or financial relationships that could be construed as a potential conflict of interest. The Reviewer MM declared a past coauthorship/collaboration with one of the authors JG.

## Publisher's Note

All claims expressed in this article are solely those of the authors and do not necessarily represent those of their affiliated organizations, or those of the publisher, the editors and the reviewers. Any product that may be evaluated in this article, or claim that may be made by its manufacturer, is not guaranteed or endorsed by the publisher.
